# Association between systemic iron status and β-cell function and insulin sensitivity in patients with newly diagnosed type 2 diabetes

**DOI:** 10.3389/fendo.2023.1143919

**Published:** 2023-04-03

**Authors:** Yao Qin, Yiting Huang, Yuxiao Li, Lu Qin, Qianying Wei, Xin Chen, Chuanhui Yang, Mei Zhang

**Affiliations:** ^1^ Department of Endocrinology, the First Affiliated Hospital of Nanjing Medical University, Nanjing, China; ^2^ Department of Clinical Nutrition, the Second Affiliated Hospital of Soochow University, Suzhou, China; ^3^ Department of Endocrinology, the First People’s Hospital of Lianyungang, Lianyungang, China

**Keywords:** type 2 diabetes, β-cell function, serum ferritin, transferrin, iron

## Abstract

**Objective:**

Abnormal iron metabolism is related to the risk of diabetes, but the underlying mechanism of this association remains uncertain. This study was conducted to evaluate the contributions of systemic iron status to β-cell function and insulin sensitivity of patients with newly diagnosed T2DM.

**Methods:**

A total of 162 patients with newly diagnosed T2DM and 162 healthy controls were enrolled in the study. Basic characteristics, biochemical indicators, and iron metabolism biomarkers, including serum iron (SI), ferritin (SF), transferrin (Trf), and transferrin saturation (TS), were collected. All patients underwent a 75 g oral glucose tolerance test. A series of parameters for assessing β-cell function and insulin sensitivity were calculated. The multivariate stepwise linear regression model was used to investigate the contributions of iron metabolism to β-cell function and insulin sensitivity.

**Results:**

Compared with healthy controls, patients with newly diagnosed T2DM had significantly higher levels of SF. Among the diabetic patients, the SI and TS levels were higher, and the percentage of Trf levels below normal values was lower in men than in women. In all diabetic patients, SF was the independent risk factor associated with impaired β-cell function. Further stratification analysis showed that Trf was an independent protective factor for β-cell function in male patients, while SF was an independent risk factor for impaired β-cell function in female patients. However, systemic iron status did not affect insulin sensitivity.

**Conclusion:**

Elevated SF levels and decreased Trf levels had a profound effect on impaired β-cell function in Chinese patients with newly diagnosed T2DM.

## 1 Introduction

Type 2 diabetes mellitus (T2DM) is a metabolic disease characterized by insulin resistance and relative insulin deficiency (1). There is a quantitative interaction between insulin action and insulin release. When insulin sensitivity decreases, changes in insulin sensitivity are compensated by an increase in insulin secretion to balance the blood glucose level (2). As the disease progresses, the number of β cells progressively decreases, and β-cell function deteriorates further, eventually resulting in insulin deficiency and obvious diabetes. However, the mechanisms that mediate insulin secretion defect and insulin resistance have not yet been fully elucidated.

The initial observation of an increased incidence of T2DM in patients with hereditary hemochromatosis and the similar discovery subsequently in secondary iron overload diseases (such as thalassemia) sparked interest in the relationship between iron metabolism and T2DM (3, 4). There is increasing evidence that iron overload is an important determinant of insulitis and a biological marker of the increased risk and mortality of diabetes (5). In type 1 and type 2 diabetes, and even among diabetic complications, most patients have abnormal iron metabolism, manifested by serum iron (SI), ferritin (SF), and transferrin saturation (TS) levels that are significantly higher than in the general population (6–8). Recently, Wang et al. used Mendelian randomization to analyze the causal relationship between systematic iron increase and the risk of T2DM from a genetic perspective. The results showed that genetically instrumented SI, SF, and TS were positively related to the risk of T2DM, while transferrin (Trf) was negatively correlated with the risk of T2DM (9). Interventions to reduce iron have been reported to delay the onset of T2DM, including the use of chelators (10, 11), blood-letting (12), and an iron restriction diet (13).

Studies in non-diabetic individuals showed that SF levels were positively correlated with homeostasis model assessment of insulin resistance (HOMA-IR) (14, 15). In a large study of 6,392 individuals in the Danish general population, elevated SF levels were related to impaired β-cell function, and an association with decreased insulin sensitivity was observed among men and older women but not among younger women (16). On the other hand, a cohort study of T2DM cases suggested that higher levels of SF and Trf were associated with a higher risk of T2DM and that elevated TS was associated with a lower risk of T2DM in women (17). Other studies also demonstrated that elevated SF was one of the risk factors for T2DM, and the soluble transferrin receptor-to-ferritin ratio was inversely related to the risk of T2DM (18, 19). Although abnormal iron metabolism is linked to an increased risk of T2DM, the exact role of iron metabolism in diabetes remains uncertain, which may be associated with pancreatic β-cell damage, insulin resistance, and liver dysfunction (20). At present, only one small sample study of adult men indicated that elevated levels of SF were positively associated with insulin resistance in newly diagnosed diabetics (21). The existing studies on the correlation between iron metabolism (mainly SF) and islet β-cell function mostly focused on non-diabetic individuals. The contribution weights of different iron metabolism indicators (including SI, SF, TS, and Trf) in decreased insulin secretion and impaired insulin sensitivity are still unclear, especially in patients with newly diagnosed T2DM. Therefore, this study aimed to assess the relative contributions of various iron status biomarkers to β-cell function and insulin sensitivity in patients with newly diagnosed T2DM.

## 2 Subjects and methods

### 2.1 Subjects

A total of 162 patients (90 men and 72 women) with newly diagnosed T2DM who were hospitalized in the Endocrinology Department of the First Affiliated Hospital of Nanjing Medical University from June 2017 to April 2020 were selected as the research subjects. All patients met the criteria for the diagnosis and classification of diabetes established by the WHO in 1999, and all patients did not receive any treatment. A total of 162 healthy controls (90 men and 72 women) from the same geographic area were selected. The inclusion criteria for the healthy controls were as follows: (1) no family history of diabetes and (2) fasting blood glucose <5.6 mmol/l and 2-h postprandial blood glucose <7.8 mmol/l on the oral glucose tolerance test. The exclusion criteria for all subjects in both groups were as follows: (1) those with severe heart, liver, or renal disease; (2) those with malignant tumors, hematologic disorders, autoimmune diseases, infectious diseases, or psychiatric disease; (3) those with chronic or acute inflammation; and (4) those who had recently received blood transfusion, iron, or hormone therapy. Written informed consent was obtained from all participants. The study was approved by the Ethics Committee of the First Affiliated Hospital of Nanjing Medical University and was conducted in accordance with the principles of the Declaration of Helsinki II.

### 2.2 Anthropometric and biochemical measurements

Anthropometric and biochemical measures were obtained after overnight fasting. Weight (kg) and height (cm) were measured with the participants in light indoor clothes and without shoes. Resting blood pressure was measured using a manual sphygmomanometer. Glucose levels, total cholesterol (TC), triglycerides (TG), HDL-C (high-density lipoprotein cholesterol), LDL-C (low-density lipoprotein cholesterol), and uric acid (UA) were measured with an automatic biochemical analysis system (Control AU5800, Beckman Coulter, Japan). HbA1c concentration was tested using high-performance liquid chromatography models (Bio-Rad Laboratories, USA). 25-hydroxyvitamin D (25(OH)D) concentration was measured using an electrochemiluminescence immunoassay on Cobas e411 Elecsys 2010 (Roche Diagnostics GmbH, Mannheim, Germany). SI and total iron-binding capacity (TIBC) were detected with a Ferrozine colorimetric assay (AU5821, Beckman Coulter, Japan). TS was calculated and expressed as a percentage (SI/TIBC × 100%). SF was measured with a chemiluminescent microparticle immunoassay (DXI800, Beckman Coulter, USA), and Trf was determined using immunoturbidimetry (IMAGE800, Beckman Coulter, USA). All diabetic patients underwent a 75 g OGTT with plasma glucose, serum insulin, and C-peptide measured at fasting and at 30, 60, 120, and 180 min after the oral glucose load. Insulin and C-peptide levels were measured with electrochemiluminescence (Cobas e602, Roche Inc., Germany). The reference range for the SI levels was between 10.7 and 32.2 μmol/l, for SF between 23.9 and 336.2 ng/ml, for Trf between 2.0 and 3.6 g/l, for TS between 20% and 55%, for TC between 3.00 and 5.70 mmol/l, for TG between 0.00 and 2.25 mmol/l, for HDL-C between 1.03 and 1.55 mmol/l, for LDL-C between 2.60 and 4.10 mmol/l, for UA between 155 and 357 μmol/l, and for 25(OH)D between 52.5 and 117.5 mmol/l.

### 2.3 Assays and calculations

Estimates of β-cell function were calculated with homeostasis model assessment of β-cell function (HOMA-β), corrected insulin response (CIR), the insulinogenic index (IGI), and the ratio of the area under the insulin curve to the area under the glucose curve (AUC_Ins_/AUC_Glu_). HOMA-β was calculated as (20 × fasting insulin (μU/ml))/(fasting glucose (mmol/l) - 3.5) (22). CIR was calculated as (100 × 30 min insulin (pmol/l))/(30 min glucose (mmol/l) × (30 min glucose (mmol/l) - 3.89)). The IGI was calculated as (30 min insulin - fasting insulin)/(30 min glucose - fasting glucose) (pmol/mmol), reflecting the early and midsecretion of insulin. AUC_Ins_/AUC_Glu_ was calculated as AUC3-h insulin/AUC3-h glucose (pmol/mmol). Insulin sensitivity was estimated using the HOMA-IR and the Matsuda index of insulin sensitivity (ISI_Matsuda_). The HOMA-IR was calculated as (fasting insulin (μU/ml) × fasting glucose (mmol/l))/22.5 (22). The ISI_Matsuda_ was calculated as 10000/(fasting glucose (mg/dl) × fasting insulin (μU/ml) × mean OGTT glucose concentration × mean OGTT insulin concentration)^1/2^ (23).

### 2.4 Statistical analysis

SPSS version 24.0 was used for the data analysis. The normality of the quantitative variables was evaluated using the *Shapiro-Wilk* test. Normally distributed continuous variables were presented as mean ± S.D., and the groups were compared with the independent sample *t* test. Non-normally distributed continuous variables were presented as median (first quartile, third quartile), and the groups were compared with the *Mann-Whitney U* test. The proportions were calculated for the categorical data, and the *Chi*-square test was used for the categorical variables. Spearman correlation analysis was used to evaluate the correlation between pancreatic β-cell function or insulin sensitivity and iron metabolism indicators and the associated factors, and multivariate stepwise linear regression analysis was used to investigate the independent effect of iron metabolism on β-cell function or insulin sensitivity (after logarithmic transformation). *P* < 0.05 was considered statistically significant.

## 3 Results

### 3.1 Patients with newly diagnosed T2DM had higher SF levels than healthy controls

The characteristics and clinical laboratory data of 162 patients with newly diagnosed T2DM and 162 healthy controls were presented in **Table 1**. The patients with T2DM had a mean age of 52.88 ± 13.63 years and a median body mass index (BMI) of 25.38 (23.44, 27.91) kg/m^2^, which were higher than in the healthy controls (*P* = 0.001, *P* = 0.000, respectively). Compared with the healthy controls, the levels of TG and HbA1c in the patients with T2DM were higher (*P* = 0.002, *P* = 0.000, respectively), while the HDL-C levels were lower (*P* = 0.000). More importantly, the SF levels were significantly higher in the patients with T2DM than in the healthy controls (*P* = 0.003). There were no significant differences in the RBC, Hb, TC, and LDL-C levels between the two groups (all *P* > 0.05).

**Table 1:** Characteristics of patients with newly diagnosed T2DM and healthy controls.

	T2DM (n = 162)	Healthy controls (n = 162)	*P*
Male (%)	90 (55.6%)	90 (55.6%)	1.000
Age (years)	52.88 ± 13.63	49.00 (45.00, 53.00)	0.001
BMI (kg/m^2^)	25.38 (23.44, 27.91)	23.82 (21.82, 25.31)	0.000
RBC (^10^12^/l)	4.74 (4.49, 5.07)	4.75 (4.55, 4.98)	0.878
Hb (g/l)	143.70 ± 14.26	144.09 ± 12.99	0.798
TC (mmol/l)	4.73 ± 1.04	4.87 (4.37, 5.37)	0.130
TG (mmol/l)	1.38 (1.07, 1.94)	1.24 (0.83, 1.68)	0.002
HDL-C (mmol/l)	1.03 (0.89, 1.16)	1.29 (1.15, 1.52)	0.000
LDL-C (mmol/l)	2.92 (2.52, 3.38)	3.02 (2.70, 3.45)	0.073
HbA1c (%)	9.45 (7.50, 11.53)	5.20 (5.10, 5.30)	0.000
SF (ng/ml)	230.80 (94.70, 448.70)	144.90 (85.25, 281.78)	0.003

Data are shown as mean ± S.D., median (25th percentile, 75th percentile), or n (%). *P* < 0.05 considered significant. T2DM, type 2 diabetes mellitus; BMI, body mass index; RBC, red blood cells; Hb, hemoglobin; TC, total cholesterol; TG, triglycerides; HDL-C, high-density lipoprotein cholesterol; LDL-C, low-density lipoprotein cholesterol; HbA1c, glycosylated hemoglobin A1c; SF, serum ferritin.

### 3.2 Iron metabolism, β-cell function, and insulin sensitivity in patients with newly diagnosed T2DM

A total of 162 patients with newly diagnosed T2DM underwent a 75 g OGTT and iron metabolism assessment. As shown in **Table 2**, the median BMI for all patients was 25.38 (23.44, 27.91) kg/m^2^ and was higher in men than in women (*P* = 0.013). The median HDL-C for all patients was 1.03 (0.89, 1.16) mmol/l and was higher in women than in men (*P* = 0.007). The median UA and 25(OH)D for all patients were 323.00 (267.00, 391.25) μmol/l and 41.20 (32.71, 55.55) mmol/; both were higher in men than in women (*P* = 0.000, *P* = 0.000, respectively). In terms of iron metabolism, the median SI for all patients was 16.10 (12.10, 20.88) μmol/l, and the median TS was 32.48 (21.16, 44.84) %; both were higher in men than in women (*P* = 0.019, *P* = 0.024, respectively). The percentage of Trf levels found to be below normal values was lower in male than in female patients (14.44% *vs.* 29.17%, *P* = 0.022). However, the percentages of elevated SI, SF, and TS levels were not statistically different between male and female patients. Regarding β-cell function and insulin sensitivity, the median ISI_Matsuda_ for all patients was 0.11 (0.05, 0.21) and was significantly lower in women than in men (*P* = 0.039). The average age for all patients was 52.88 ± 13.63 years, and the median HbA1c was 9.45 (7.50, 11.53) %. The mean TC for all patients was 4.73 ± 1.04 mmol/l, the median TG was 1.38 (1.07, 1.94) mmol/l, and the median LDL-C was 2.92 (2.52, 3.38) mmol/l. The median SF for all patients was 230.80 (94.70, 448.70) ng/ml, and the mean Trf was 2.38 ± 0.54 g/l. The median HOMA-β for all patients was 43.93 (22.36, 78.94), the median CIR was 179.05 (90.84, 412.69), the median IGI was 17.49 (8.50, 37.70), and the median AUC_Ins_/AUC_Glu_ was 12.39 (6.58, 24.82). The median HOMA-IR for all patients was 1.49 (0.70, 2.68). There were no significant differences in age, SBP, DBP, HbA1c, TC, TG, LDL-C, SF, or Trf levels between the two groups, or in β-cell function and HOMA-IR (all *P* > 0.05).

**Table 2:** Clinical characteristics, β-cell function and insulin sensitivity of patients with newly diagnosed T2DM.

	All (n = 162)	Male (n = 90)	Female (n = 72)	*P*
Age (years)	52.88 ± 13.63	50.74 ± 14.32	55.54 ± 12.30	0.294
BMI (kg/m^2^)	25.38 (23.44, 27.91)	26.43 (24.12, 28.39)	24.34 (22.97, 27.65)	0.013
SBP (mmHg)	133.40 ± 17.83	131.41 ± 16.32	135.89 ± 19.38	0.112
DBP (mmHg)	79.46 ± 12.62	80.21 ± 12.14	78.51 ± 13.21	0.397
HbA1c (%)	9.45 (7.50, 11.53)	9.50 (7.50, 11.83)	9.30 (7.93, 11.25)	0.653
TC (mmol/l)	4.73 ± 1.04	4.69 ± 1.00	4.77 ± 1.10	0.394
TG (mmol/l)	1.38 (1.07, 1.94)	1.40 (1.10, 1.86)	1.35 (1.03, 2.08)	0.705
HDL-C (mmol/l)	1.03 (0.89, 1.16)	0.99 ± 0.19	1.07 (0.91, 1.26)	0.007
LDL-C (mmol/l)	2.92 (2.52, 3.38)	2.97 ± 0.76	3.01 ± 0.79	0.564
UA (μmol/l)	323.00 (267.00, 391.25)	346.00 (281.50, 413.25)	302.58 ± 86.47	0.000
25(OH)D (mmol/l)	41.20 (32.71, 55.55)	44.05 (35.53, 63.75)	34.47 (29.08, 45.90)	0.000
Iron metabolism				
SI (μmol/l)	16.10 (12.10, 20.88)	17.55 (13.40, 21.13)	13.60 (11.40, 19.88)	0.019
Elevated SI [n (%)]	1 (0.62)	1 (1.11)	0 (0.00)	0.370
SF (ng/ml)	230.80 (94.70, 448.70)	243.50 (101.38, 403.38)	190.65 (88.18, 586.73)	0.627
Elevated SF [n (%)]	57 (35.19)	30 (33.33)	27 (37.50)	0.581
Trf (g/l)	2.38 ± 0.54	2.49 ± 0.50	2.24 ± 0.56	0.376
Decreased Trf [n (%)]	34 (20.99)	13 (14.44)	21 (29.17)	0.022
TS (%)	32.48 (21.16, 44.84)	34.10 (24.04, 45.47)	25.65 (18.80, 41.13)	0.024
Elevated TS [n (%)]	27 (16.67)	14 (15.56)	13 (18.06)	0.671
β-cell function				
HOMA-β	43.93 (22.36, 78.94)	40.55 (23.26, 77.72)	46.33 (20.29, 79.16)	0.917
CIR	179.05 (90.84, 412.69)	176.02 (95.23, 402.93)	185.79 (88.49, 418.95)	0.995
IGI	17.49 (8.50, 37.70)	16.66 (7.76, 33.56)	18.80 (9.36, 42.84)	0.174
AUC_Ins_/AUC_Glu_	12.39 (6.58, 24.82)	11.97 (6.58, 19.63)	13.28 (6.54, 31.71)	0.363
Insulin sensitivity				
HOMA-IR	1.49 (0.70, 2.68)	1.32 (0.60, 2.58)	1.84 (0.84, 2.70)	0.148
ISI_Matsuda_	0.11 (0.05, 0.21)	0.14 (0.05, 0.26)	0.09 (0.04, 0.18)	0.039

Data are shown as mean ± S.D. or median (25th percentile, 75th percentile). P value reports the difference between male and female patients, *P* < 0.05 considered significant. BMI, body mass index; SBP, systolic blood pressure; DBP, diastolic blood pressure; HbA1c, glycosylated hemoglobin A1c; TC, total cholesterol; TG, triglycerides; HDL-C, high-density lipoprotein cholesterol; LDL-C, low-density lipoprotein cholesterol; UA, uric acid; 25(OH)D, 25-hydroxyvitamin D; SI, serum iron; SF, serum ferritin; Trf, transferrin; TS, transferrin saturation; HOMA-β, homeostasis model assessment of β-cell function; CIR, corrected insulin response; IGI, insulinogenic index; AUC_Ins_/AUC_Glu_, the ratio of the area under the insulin curve to the area under the glucose curve; HOMA-IR, homeostasis model assessment of insulin resistant; ISI_Matsuda_, the Matsuda index of insulin sensitivity.

### 3.3 Correlation between β-cell function or insulin sensitivity and systemic iron status and the associated factors in patients with newly diagnosed T2DM

**Figure 1:**
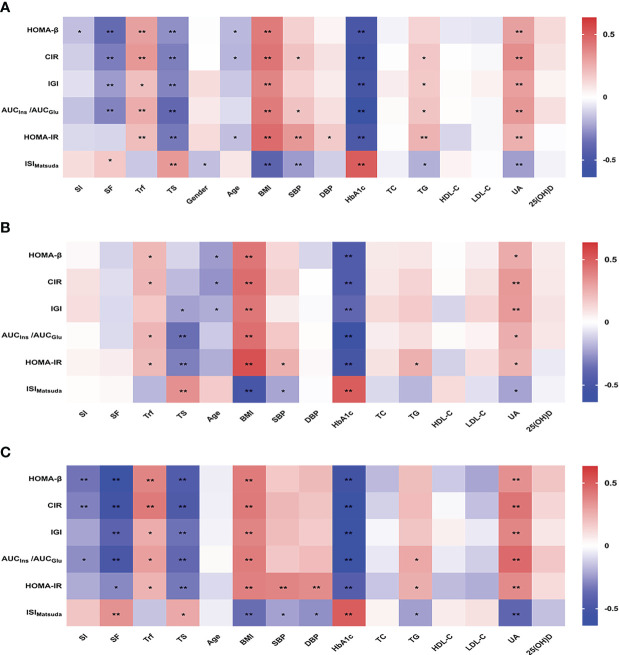
Heatmap showing *Spearman* correlation between β-cell function or insulin sensitivity and systemic iron status and the associated factors in all **(A)**, male **(B)** and female **(C)** patients with newly diagnosed T2DM. Red indicates positive correlations and blue negative. *P<0.05; **P<0.01. SI, serum iron; SF, serum ferritin; Trf, transferrin; TS, transferrin saturation; BMI, body mass index; SBP, systolic blood pressure; DBP, diastolic blood pressure; HbA1c, glycosylated hemoglobin A1c; TC, total cholesterol; TG, triglycerides; HDL-C, high-density lipoprotein cholesterol; LDL-C, low-density lipoprotein cholesterol; UA, uric acid; 25(OH)D, 25-hydroxyvitamin D; HOMA-β, homeostasis model assessment of β-cell function; CIR, corrected insulin response; IGI, insulinogenic index; AUC_Ins_/AUC_Glu_, the ratio of the area under the insulin curve to the area under the glucose curve; HOMA-IR, homeostasis model assessment of insulin resistant; ISI_Matsuda_, the Matsuda index of insulin sensitivity.

We further analyzed the correlation between β-cell function or insulin sensitivity and systemic iron status and the related factors in the patients with newly diagnosed T2DM. A heat map of the *Spearman* correlation showed a significant correlation between β-cell function or insulin sensitivity and iron status parameters and other associated factors (**Figure 1**). In all patients (**Figure 1A**), β-cell function was negatively associated with SI, SF, TS, age, and HbA1c, and positively associated with Trf, BMI, SBP, TG, and UA (all *P* < 0.05). Insulin sensitivity was positively related to SF, TS, age, and HbA1c, and negatively related to Trf, gender, BMI, SBP, DBP, TG, and UA (all *P* < 0.05). Next, we performed a stratified analysis according to sex. In male patients (**Figure 1B**), β-cell function was negatively correlated with TS, age, and HbA1c, and positively correlated with Trf, BMI, and UA (all *P* < 0.05). Insulin sensitivity was positively related to TS and HbA1c, and negatively related to Trf, BMI, SBP, TG, and UA (all *P* < 0.05). However, SI and SF were not significantly associated with β-cell function or insulin sensitivity. On the other hand, in female patients (**Figure 1C**), β-cell function was negatively associated with SI, SF, TS, and HbA1c, and positively associated with Trf, BMI, TG, and UA (all *P* < 0.05). Insulin sensitivity was positively related to SF, TS, and HbA1c, and negatively related to Trf, BMI, SBP, DBP, TG, and UA (all *P* < 0.05). Overall, the associations of all four iron biomarkers with β-cell function or insulin sensitivity were stronger among women than among men.

### 3.4 Multivariate stepwise regression analysis for islet β-cell function or insulin sensitivity and systemic iron status

We next conducted multivariate stepwise regression analysis to determine the independent factors affecting pancreatic β-cell function or insulin sensitivity in patients with newly diagnosed diabetes. We examined the relationship between the natural log (Ln)-transformed β-cell function or insulin sensitivity and several statistically significant variables, including gender, age, BMI, SBP, DBP, HbA1c, TG, UA, SI, SF, Trf, and TS. In all patients (**Table 3**), SF was an independent risk factor associated with impaired β-cell function. Expressed per unit change, an increase of 1 ng/ml in SF was associated with a decrease of 1 in HOMA-β (*B* = -0.001, *P* = 0.000), a decrease of 1 in CIR (*B* = -0.001, *P* = 0.001), and a decrease of 1 in AUC_Ins_/AUC_Glu_ (*B* = -0.001, *P* = 0.005). Subsequently, stratified analyses were performed by gender. In male patients (**Table 4**), an increase of 1 g/l in Trf was associated with an increase of 1.52 in HOMA-β (*B* = 0.416, *P* = 0.018) and an increase of 1.56 in CIR (*B* = 0.447, *P* = 0.013). However, in female patients (**Table 5**), an increase of 1 ng/ml in SF was associated with a decrease of 1 in HOMA-β (*B* = -0.001, *P* = 0.000), a decrease of 1 in CIR (*B* = -0.001, *P* = 0.002), and a decrease of 1 in AUC_Ins_/AUC_Glu_ (*B* = -0.001, *P* = 0.002). However, no correlation was found between insulin sensitivity and iron metabolism indicators in male and female patients.

**Table 3:** Multivariate stepwise regression analysis for β-cell function or insulin sensitivity and related factors in all diabetic patients.

Dependent variables	Model	*B*	*SE*	*β*	*t*	*P*
Ln (HOMA-β)	Constant	3.952	0.628	—	6.297	0.000
	HbA1c	-0.116	0.025	-0.298	-4.641	0.000
	BMI	0.069	0.016	0.288	4.371	0.000
	SF	-0.001	0.000	-0.311	-4.960	0.000
	Age	-0.010	0.005	-0.132	-2.071	0.040
Ln (CIR)	Constant	5.074	0.520	—	9.763	0.000
	HbA1c	-0.174	0.025	-0.434	-7.007	0.000
	BMI	0.053	0.016	0.216	3.279	0.001
	SF	-0.001	0.000	-0.204	-3.372	0.001
	UA	0.002	0.001	0.198	3.108	0.002
Ln (IGI)	Constant	2.668	0.615	—	4.334	0.000
	HbA1c	-0.197	0.029	-0.448	-6.727	0.000
	BMI	0.054	0.019	0.199	2.782	0.006
	UA	0.002	0.001	0.171	2.462	0.015
Ln (AUC_Ins_/AUC_Glu_)	Constant	2.399	0.423	—	5.665	0.000
	HbA1c	-0.169	0.020	-0.489	-8.371	0.000
	BMI	0.055	0.013	0.259	4.184	0.000
	UA	0.002	0.001	0.175	2.912	0.004
	SF	-0.001	0.000	-0.163	-2.864	0.005
Ln (HOMA-IR)	Constant	-1.280	0.551	—	-2.322	0.021
	BMI	0.111	0.016	0.441	6.828	0.000
	HbA1c	-0.135	0.026	-0.332	-5.137	0.000
Ln (ISI_Matsuda_)	Constant	0.521	0.706	—	0.738	0.462
	BMI	-0.104	0.019	-0.359	-5.368	0.000
	HbA1c	0.170	0.029	0.363	5.851	0.000
	Gender	-0.645	0.152	-0.270	-4.251	0.000
	UA	-0.002	0.001	-0.202	-3.014	0.003

B, partial regression coefficient for the constant; SE, the standard error around the coefficient for the constant; β, standard partial regression coefficient; Ln, natural log-transformed; HOMA-β, homeostasis model assessment of β-cell function; CIR, corrected insulin response; IGI, insulinogenic index; AUC_Ins_/AUC_Glu_, the ratio of the area under the insulin curve to the area under the glucose curve; HOMA-IR, homeostasis model assessment of insulin resistant; ISI_Matsuda_, the Matsuda index of insulin sensitivity; HbA1c, glycosylated hemoglobin A1c; BMI, body mass index; SF, serum ferritin; UA, uric acid.

**Table 4:** Multivariate stepwise regression analysis for β-cell function or insulin sensitivity and related factors in men.

Dependent variables	Model	*B*	*SE*	*β*	*t*	*P*
Ln (HOMA-β)	Constant	2.545	0.984	—	2.586	0.012
	HbA1c	-0.125	0.041	-0.335	-3.060	0.003
	Trf	0.416	0.171	0.250	2.430	0.018
	BMI	0.058	0.025	0.240	2.310	0.024
Ln (CIR)	Constant	5.252	0.722	—	7.274	0.000
	HbA1c	-0.202	0.039	-0.477	-5.122	0.000
	UA	0.003	0.001	0.250	2.820	0.006
	Trf	0.447	0.175	0.237	2.563	0.013
Ln (IGI)	Constant	4.248	0.535	—	7.937	0.000
	HbA1c	-0.243	0.037	-0.580	-6.577	0.000
	UA	0.003	0.001	0.285	3.229	0.002
Ln (AUC_Ins_/AUC_Glu_)	Constant	3.450	0.429	—	8.032	0.000
	HbA1c	-0.195	0.030	-0.562	-6.580	0.000
	UA	0.004	0.001	0.349	4.081	0.000
Ln (HOMA-IR)	Constant	-0.214	0.875	—	-0.245	0.808
	HbA1c	-0.163	0.041	-0.403	-3.936	0.000
	SBP	0.016	0.005	0.311	3.040	0.003
Ln (ISI_Matsuda_)	Constant	-3.028	0.664	—	-4.560	0.000
	HbA1c	0.193	0.046	0.421	4.214	0.000
	UA	-0.004	0.001	-0.329	-3.293	0.000

B, partial regression coefficient for the constant; SE, the standard error around the coefficient for the constant; β, standard partial regression coefficient; Ln, natural log-transformed; HOMA-β, homeostasis model assessment of β-cell function; CIR, corrected insulin response; IGI, insulinogenic index; AUC_Ins_/AUC_Glu_, the ratio of the area under the insulin curve to the area under the glucose curve; HOMA-IR, homeostasis model assessment of insulin resistant; ISI_Matsuda_, the Matsuda index of insulin sensitivity; HbA1c, glycosylated hemoglobin A1c; Trf, transferrin; BMI, body mass index; UA, uric acid; SBP, systolic blood pressure. *t*: Significance of independent variable. — means that the standard partial regression coefficient is not valid for the constant.

**Table 5:** Multivariate stepwise regression analysis for β-cell function or insulin sensitivity and related factors in women.

Dependent variables	Model	*B*	*SE*	*β*	*t*	*P*
Ln (HOMA-β)	Constant	4.747	0.468	—	10.141	0.000
	SF	-0.001	0.000	-0.479	-5.286	0.000
	HbA1c	-0.116	0.034	-0.309	-3.383	0.001
	UA	0.002	0.001	0.185	2.127	0.037
Ln (CIR)	Constant	6.539	0.526	—	12.439	0.000
	HbA1c	-0.195	0.038	-0.460	-5.071	0.000
	SF	-0.001	0.000	-0.293	-3.252	0.002
	UA	0.003	0.001	0.247	2.864	0.006
Ln (IGI)	Constant	4.248	0.535	—	7.937	0.000
	HbA1c	-0.243	0.037	-0.580	-6.577	0.000
	UA	0.003	0.001	0.285	3.229	0.002
Ln (AUC_Ins_/AUC_Glu_)	Constant	3.491	0.404	—	8.647	0.000
	HbA1c	-0.164	0.029	-0.474	-5.578	0.000
	UA	0.003	0.001	0.323	4.008	0.000
	SF	-0.001	0.000	-0.269	-3.186	0.002
Ln (HOMA-IR)	Constant	-0.456	0.857	—	-0.533	0.596
	HbA1c	-0.128	0.043	-0.316	-2.972	0.004
	SBP	0.018	0.005	0.336	3.359	0.001
Ln (ISI_Matsuda_)	Constant	-3.028	0.664	—	-4.560	0.000
	HbA1c	0.193	0.046	0.421	4.214	0.000
	UA	-0.004	0.001	-0.329	-3.293	0.002

B, partial regression coefficient for the constant; SE, the standard error around the coefficient for the constant; β, standard partial regression coefficient; Ln, natural log-transformed; HOMA-β, homeostasis model assessment of β-cell function; CIR, corrected insulin response; IGI, insulinogenic index; AUC_Ins_/AUC_Glu_, the ratio of the area under the insulin curve to the area under the glucose curve; HOMA-IR, homeostasis model assessment of insulin resistant; ISI_Matsuda_, the Matsuda index of insulin sensitivity; SF, serum ferritin; HbA1c, glycosylated hemoglobin A1c; UA, uric acid; SBP, systolic blood pressure.

## 4 Discussion

In this study, we analyzed the SF levels of patients with newly diagnosed T2DM and healthy controls, and the correlation between pancreatic β-cell function or insulin sensitivity and systemic iron status (reflected by the four iron biomarkers) in patients with newly diagnosed T2DM. The study provides evidence that SF levels (a marker of iron stores) in patients with newly diagnosed T2DM were significantly higher than in healthy controls, and the SI and TS levels were higher in male than in female diabetic patients. Multiple stepwise regression analysis suggested that Trf was an independent protective factor against β-cell functional impairment in male patients with newly diagnosed T2DM, while SF was an independent risk factor in female patients. Systemic iron status did not independently affect insulin sensitivity.

T2DM is a progressive metabolic disorder characterized by insulin resistance and β-cell dysfunction. The onset of β-cell dysfunction has been considered a late event in the pathogenesis of diabetes. In a previous study, we showed that the median level of HOMA-β was 103.56 in Chinese healthy controls (24). In this study, HOMA-β was found to be significantly decreased in patients with newly diagnosed T2DM, suggesting that the decline in β-cell function may be an early event. Whether impaired β-cell function at an early stage is associated with abnormal iron metabolism is unknown. Other studies describing the correlation between iron metabolism and T2DM were inconsistent among genders and races. SF concentrations were significantly higher in women with diabetes than in women without diabetes in all racial/ethnic groups, but the concentrations were significantly lower in Asian men with diabetes than in those without diabetes (25). In this study, the SF concentrations were significantly higher in the patients with newly diagnosed T2DM than in the healthy controls, but there was no difference in SF levels between men and women. In addition, the median levels of SI, Trf, and TS in patients with newly diagnosed T2DM were in the normal range, but the levels of SI and TS were higher in men than in women, and the percentage of Trf levels below normal values was lower in male than in female patients. Therefore, abnormal iron metabolism in patients with newly diagnosed T2DM is characterized by increased SF levels, suggesting that iron storage is increased at the early stage of T2DM.

SF levels were elevated in patients with T2DM, and increased SF levels were associated with an increased risk of diabetes (18). However, whether this association can be explained by impaired β-cell function, reduced insulin sensitivity, or both has not been fully confirmed. Recently, a small sample study of adult men showed that SF and hepcidin (the central regulator of iron homeostasis, which can regulate plasma iron concentrations) levels were significantly increased in patients with newly diagnosed T2DM, and there was a positive correlation between SF levels and HOMA-IR (21). Consistent with the previous study, we also observed that the SF levels in patients with newly diagnosed T2DM were significantly higher than in healthy controls, suggesting that patients with T2DM may have iron overload. Further multivariate stepwise regression analysis showed that SF was an independent risk factor for impaired β-cell function in female patients with newly diagnosed T2DM, indicating that increased iron storage might interact with other genetic and environmental factors, thus impairing β-cell function and affecting insulin secretion. However, the SF levels were not related to insulin sensitivity. There are several possible reasons for the discrepancy between the results of this study and the previous publication, including differences in the ethnicity of the present study’s population, sample size, and functional features of β-cell at the time of diabetes diagnosis.

Trf, another marker of iron status, is the major serum iron-binding and -transport protein (26). Increased systemic iron status has been associated with increased levels of SI, SF, and TS, as well as decreased levels of Trf (27). It has been proposed that Trf is an antioxidant, as it binds and prevents free iron from participating in the Fenton reaction (28). Genetically instrumented Trf was inversely associated with T2DM in the Chinese population (9), and a low serum Trf concentration was associated with diabetic end-stage renal disease in Chinese patients with T2DM (29). However, an increase in SF and Trf predicted insulin resistance and new onset T2DM in non-T2DM subjects in a European population (17, 30, 31), and Trf was positively associated with incident T2DM in Koreans (32). Thus, the results of previous studies were contradictory. In this study, we analyzed the correlation between Trf and β-cell function or insulin sensitivity, and demonstrated that Trf exerted an independent protective effect on β-cell function in male patients with newly diagnosed T2DM.

TS levels reflect the levels of non-transferrin-bound iron, which is considered an important source of iron deposition and toxicity in organs (33, 34). However, the results from existing perspective studies on the relationship between TS and T2DM are contradictory. A study that used data from the National Health and Nutrition Examination Survey did not find any association between TS and T2DM (35). A study performed in men showed that TS levels were similar in diabetics, prediabetics, and control subjects (21). In contrast, a meta-analysis of three Danish studies found that TS ≥50% was associated with a higher risk of T2DM (36). However, a prospective study showed that elevated TS levels are associated with a lower risk of T2DM, which was statistically significant only in women (17). In the present study, bivariate correlational analysis revealed a negative correlation of TS with β-cell function and a positive association with insulin sensitivity, but these correlations were not observed in multiple stepwise regression analysis. The results suggest that the association between increased iron absorption or higher non-transferrin-bound iron and T2DM is complex. Therefore, large-scale population-based prospective studies are needed to elucidate this issue.

Free iron has the ability to generate reactive oxygen species, which may lead to increased oxidative stress and cell damage. Therefore, excessive free iron may be potentially hazardous (37). Pancreatic β cells are vulnerable to oxidative stress because their antioxidant defense mechanisms are particularly weak (38). Although adequate iron is critical to normal β-cell function and glucose homeostasis, studies have suggested that excessive iron may disrupt glucose homeostasis through several potential mechanisms. For example, oxidative stress caused by excessive iron accumulation leads to β-cell damage and apoptosis, resulting in reduced insulin secretion (39). However, high iron storage in the liver may induce insulin resistance by impairing insulin signal transduction and attenuating the liver’s ability to extract insulin (40). In the present study, we found that iron overload had a more profound effect on β-cell function, rather than insulin sensitivity. We speculated that the precise effect of iron overload on pancreatic β cells may depend largely on the main site of iron accumulation, which needs to be verified in animal models in the future.

The molecular mechanisms underlying the observed associations of impaired β-cell function with iron status are now being elucidated. The direct consequence of intracellular iron overload is that its entry into mitochondria can depolarize the organelle membrane potential, which affects the electron transport chain and the energy supply of insulin release (41, 42). In addition, iron and the iron-sulfur (Fe-S) cluster influence each other, causing mitochondrial iron accumulation, more reactive oxygen species (ROS) production, endoplasmic reticulum (ER) stress, failure in biosynthesis of insulin, and ferroptosis in β-cells (43). In addition, ROS directly impair insulin synthesis and secretion during the development of T2DM (44). Another mechanism by which iron overload may affect β-cell function and survival is through amylin. Heme can bind to amylin to form a complex, which leads to the formation of H_2_O_2_ *via* oxidative stress (45, 46), thus promoting ROS-mediated β-cell failure. Recently, Stancic et al. also demonstrated that high glucose, H_2_O_2_, and streptozotocin (STZ) caused the accumulation of lipofuscin, which is formed due to iron-catalyzed oxidative processes, and serves as a reservoir of metal ions (including iron), releases in its reactive form and thus promotes ROS generation and consequently β-cell ferroptosis (47). Taken together, iron accumulation is associated with insulin secretion dysfunction in pancreatic β cells.

The strength of this study is that we focused on patients with newly diagnosed T2DM and evaluated the relative contributions of systemic iron status (including SI, SF, Trf, and TS) to pancreatic β-cell function and insulin sensitivity. However, several limitations must be acknowledged. First, this was a single-center, retrospective study, in which we were unable to establish a definitive causal connection between increased SF levels and impaired β-cell function. Second, the relatively small sample size might not provide sufficient statistical power to determine small-scale associations and potential interactions; thus, a population-based prospective cohort study with a large sample size is needed. Finally, the study was conducted among Chinese populations, and future studies in more racially or ethnically diverse populations are warranted.

## 5 Conclusions

In summary, this is the first study on the relationship between systemic iron metabolism and β-cell function and insulin sensitivity in Chinese patients with newly diagnosed T2DM. We observed that SF levels in patients with newly diagnosed T2DM were significantly increased, and the SI and TS levels were higher in male patients than in female patients, although the median concentrations of SI and TS were in the normal range. We then demonstrated that Trf was an independent protective factor against β-cell functional impairment in male patients with newly diagnosed T2DM, while SF was an independent risk factor in women. Future studies should focus on demonstrating this relationship in various populations with different ethnic backgrounds, as well as the possible potential mechanisms, thus providing new insights into underlying strategies for the prevention and treatment of T2DM.

## Data availability statement

The raw data supporting the conclusions of this article will be made available by the authors, without undue reservation.

## Ethics statement

The studies involving human participants were reviewed and approved by the First Affiliated Hospital of Nanjing Medical University. The patients/participants provided their written informed consent to participate in this study.

## Author contributions

Conceptualization, MZ; methodology, LQ, QW, XC and CY; formal analysis and data curation, YQ and YH; Writing - original draft preparation, YQ and YL; Writing - review & editing, MZ; funding acquisition, YQ and MZ. All authors contributed to the article and approved the submitted version. 
